# Associations between daily home blood pressure measurements and self-reports of lifestyle and symptoms in primary care: the PERHIT study

**DOI:** 10.1080/02813432.2024.2332745

**Published:** 2024-03-26

**Authors:** Ulrika Andersson, Peter M. Nilsson, Karin Kjellgren, Mikael Ekholm, Patrik Midlöv

**Affiliations:** a Department of Clinical Sciences Malmö, Center for Primary Health Care Research, Lund University, Malmö, Sweden; b Department of Clinical Sciences Malmö, Lund University, Malmö, Sweden; c University of Gothenburg Centre for Person-Centred Care (GPCC), Sahlgrenska Academy, University of Gothenburg, Gothenburg, Sweden; d Department of Health, Medicine and Caring Sciences, Linköping University, Linköping, Sweden; e Wetterhälsan Primary Health Care Centre, Jönköping, Region Jönköping County, Sweden; f Department of Clinical Sciences, Danderyd Hospital, Karolinska Institute, Stockholm, Sweden

**Keywords:** Blood pressure, home monitoring, hypertension, primary health care, self-management

## Abstract

**Objective:**

To explore in a primary care setting the associations between patients’ daily self-measured blood pressure (BP) during eight weeks and concurrent self-reported values of wellbeing, lifestyle, symptoms, and medication intake. We also explore these associations for men and women separately.

**Design and setting:**

The study is a secondary post-hoc analysis of the randomised controlled trial PERson-centeredness in Hypertension management using Information Technology (PERHIT). The trial was conducted in primary health care in four regions in Southern Sweden.

**Patients:**

Participants (*n* = 454) in the intervention group in the PERHIT-trial used an interactive web-based system for self-management of hypertension for eight consecutive weeks. Each evening, participants reported in the system their wellbeing, lifestyle, symptoms, and medication adherence as well as their self-measured BP and heart rate.

**Main outcome measures:**

Association between self-reported BP and 10 self-report lifestyle-related variables.

**Results:**

Self-reported less stress and higher wellbeing were similarly associated with BP, with 1.0 mmHg lower systolic BP and 0.6/0.4 mmHg lower diastolic BP (*p* < 0.001). Adherence to medication had the greatest impact on BP levels (5.2/2.6 mmHg, *p* < 0.001). Restlessness and headache were also significantly associated with BP, but to a lesser extent. Physical activity was only significantly associated with BP levels for men, but not for women.

**Conclusion:**

In hypertension management, it may be important to identify patients with high-stress levels and low wellbeing. The association between medication intake and BP was obvious, thus stressing the importance of medication adherence for patients with hypertension.

## KEY POINTS

- Associations between daily home blood pressure (BP) and self-reports of lifestyle and symptoms have not been previously well explored.
- Self-reported higher wellbeing, lower restlessness, less stress, and higher medication adherence were significantly associated with lower same-day BP levels.
- Physical activity was significantly associated with same-day BP for men, but not for women.
- Using a hypertension management system may be a valuable tool for communication between the patient and physician.

## Introduction

Hypertension is one of the most common clinical diagnoses in primary health care and the most important modifiable risk factor for cardiovascular disease [1]. As a chronic condition, hypertension requires long-term commitment by the patient for successful treatment and blood pressure (BP) control [2]. Adherence to antihypertensive treatment is often suboptimal, and one reason for this may be the lack of noticeable physical feedback effect of the treatment for the patients [3]. Hypertension is often regarded as a symptomless condition, but patients’ knowledge of hypertension, and their perception about the condition and its symptoms, differ [4]. For successful hypertension management, it may be important for clinicians to consider the patient’s experiences and perspective on the condition and the treatment [5, 6]. Adopting a person-centred approach, acknowledging the patient’s beliefs and values, is considered fundamental to the care process [7]. Studies describing interventions using self-monitoring of BP show positive results on BP levels [8], indicating that the act of measuring and observing the BP values could affect treatment adherence, regardless of whether it is due to lifestyle changes or medication intake [9]. However, self-monitoring on its own does not seem to affect BP. A systematic review has concluded that self-monitoring is effective in lowering BP only when combined with additional interventions, such as counselling, education, or medication titration [10].

The current study is part of a research project entitled PERson-centredness in Hypertension management using Information Technology (PERHIT), a large randomised controlled trial performed in Swedish primary health care. In PERHIT, we tested the effect of person-centred care with an interactive web-based system for self-management of hypertension [11]. A positive effect was seen on the proportion of patients with a BP lower than 140/90 mmHg after eight weeks, but the long-term effect of the intervention was uncertain [12]. The web-based system was created in collaboration with patients and physicians, nurses, and pharmacists and based on person-centred care [13, 14]. The study was designed to promote increased hypertension awareness, knowledge, and motivation to adherence to treatment, by allowing patients to explore their daily BP and its relationship to same-day self-reported lifestyle factors, symptoms, and medication adherence. Daily self-reporting is seldom used but may be a reliable way to assess the effects of treatment, since it is easier to remember symptoms and activities on the same day. The usefulness of the system depends on discernible links between BP and self-reports. A previous analysis of data from a pilot study with 50 patients with hypertension showed associations between patients’ BP levels and self-reports of medication intake, wellbeing, stress, and physical activity [15].

The aim of this sub-study was to explore associations between patients’ daily self-measured BP and concurrent self-reported values of wellbeing, lifestyle, symptoms, and medication intake in primary care. We also aimed to explore associations for men and women separately.

## Methods

### Study design and participants

This study is an observational, secondary analysis of the randomised controlled trial PERHIT [9]. The aim of the main trial was to increase the proportion of patients with hypertension in primary care with a BP lower than 140/90 mmHg by using e-health technology and a person-centred approach. The trial has been described in previous publications [11, 12]. Adult patients with a diagnosis of hypertension and treated with at least one antihypertensive drug were recruited by nurses or physicians from 31 different primary health care centres (PHCC) in four regions of southern Sweden. They were excluded from participating in the study if they had secondary hypertension, terminal illness, pregnancy-induced hypertension, cognitive impairment, impaired vision (as they needed to be able to read text on a mobile phone) or severe mental illness.

The study period was one year, and all the participants visited their PHCC three times during this period. At the first visit, a baseline assessment was conducted, and participants were randomised 1:1 to either the intervention or the control group. Follow-up visits were scheduled after eight weeks and after one year. At each visit, office BP and heart rate were measured, blood samples were taken, and the patient filled in several questionnaires.

The participants in this sub-study were those who were included in the intervention group in the PERHIT trial. After the first study visit, the participants in the intervention group installed a program on their mobile phone called CQ (developed by Circadian Questions AB). They were also given a BP monitor (Microlife BP A6 BT) and instructed by their nurse or physician on how to use it properly at home. Participants were requested to self-report in CQ every evening for eight consecutive weeks. When they started CQ on their mobile phone, the questions were displayed in sequential order. These questions were about *quality of life* (wellbeing, stress, sleep, restlessness, and tiredness), *adherence* (medication intake), *lifestyle* (physical activity), and *symptoms* (dizziness, headache, and palpitations). Participants chose their answers on a five-stage Likert scale for the quality of life, lifestyle, and symptom reports. The question regarding medication intake had three options: yes, no, or partly. The questions and response options used in this study were developed and evaluated in the pilot study [13, 14] and are shown in Table 1.

**Table 1.** Questions and response scales in CQ.

Item	Question*	Response format (steps)
Wellbeing	How do you feel today?	Very bad – very good (5)
Medication intake	Taken your BP medicine today?	Yes – some of it – no (3)
Tiredness	Tired today?	Very much – not at all (5)
Dizziness	Dizzy today?	Very much – not at all (5)
Headache	Headache today?	Very much – not at all (5)
Palpations	Heart palpitations today?	Very much – not at all (5)
Restlessness	Restless today?	Very much – not at all (5)
Sleep	How did you sleep last night?	Very bad – very good (5)
Physical activity	Physically active today?	Very much – not at all (5)
Stress	Felt stressed today?	Very much – not at all (5)
Systolic BP	Systolic (top) blood pressure?	Value
Diastolic BP	Diastolic (bottom) blood pressure?	Value
Pulse	Pulse today?	Value

* The questions were short to fit displays of different types of mobile phones. Additional information about the questions was available for the participants in the information material handed out at the first visit. Questions are translated from Swedish. BP: blood pressure.

After answering the questions, the participants continued to measure their BP and heart rate and reported the values in CQ. The system was designed in such a way that the BP and heart rate values were not automatically transferred from the BP monitor to the program on the mobile phone. We reasoned that by actively reporting the data in the system, participants were more likely to reflect on their BP values. Of course, this design meant that the self-reports of BP and heart rate could be inaccurate. In the pilot study, the self-reported BP values were validated against the data stored in the BP monitor and only a small proportion differed [15]. The self-reported BP data were therefore considered reliable.

The self-reported data was transferred to a secure database; no data were saved on the participants’ mobile phone. Participants could access their reported values, visualized in graphs, by logging in to a secure website. By combining, for example, systolic BP (SBP) and physical activity in the same graph, participants could explore which factors affected their BP. The study staff also had access to the website during the intervention period and could use it to monitor the patient’s BP. When the intervention was completed after eight weeks, a follow-up visit was scheduled at the PHCC. For the participants in the intervention group, this was an opportunity to discuss their BP levels with their nurse or physician, and the professionals were encouraged by the research team to use the graphs as a basis for discussion. As previously presented, the use of the system enabled the patients to become more active and involved in their own treatment, and the study staff perceived it as a resource for communication about BP and lifestyle [16, 17].

### Statistical analysis

Demographic and clinical variables of participants were characterised using descriptive statistics. Variables were compared between men and women using Student’s t-test for continuous variables and Pearson’s chi^2^-test for categorical variables.

Individuals were included if they had registered at least 10 sets of reports in the system during the intervention period. The variables included in the analysis were SBP and diastolic BP (DBP) as dependent variables and self-reports of variables of quality of life, adherence, lifestyle, and symptoms as independent variables.

To predict the effect of self-reported variables on SBP and DBP, repeated-measures linear mixed-effect models were used. This method was chosen as it allows inclusion of dependent observations on repeated measurements and independent variables in the same model, thus considering possible associations between the independent variables. The self-reported variables were included as fixed effects and the participants as random effects. All 10 self-reported variables were included in the same model for SBP and DBP, respectively. The correlation structure was set to AR(1) to account for the autoregressive pattern of the residuals in the repeated measures design. Statistical significance was set to *p*-value <0.05 for all significance tests.

Statistical analyses were performed in R version 4.1.2 and RStudio version 2022.2.2.485 [18, 19].

### Ethical considerations

The PERHIT-project was approved by the Regional Ethics Review Board in Lund (2017/311 and 2019/00036). The study was registered with ClinicalTrials.gov [NCT03554382].

## Results

In the PERHIT-trial, a total of 482 individuals were included in the intervention group. In all, 28 participants registered less than 10 sets of reports during the intervention period and were therefore excluded from the analysis. Demographic and clinical variables for the included participants are described in Table 2. The variables are also reported separately for men and women. There were some differences between the sexes. Office DBP at baseline was significantly lower for women than men (*p* < 0.001) and cholesterol was significantly higher for women than men (*p* < 0.001). More men than women were treated with calcium channel blockers (*p* < 0.001). Men reported higher alcohol consumption than women (*p* = 0.004). There were also significant differences between men and women regarding marital status, educational level, and occupation, as more men were married (*p* = 0.025), more women had university-level education (*p* < 0.001), and men were working to a greater extent (*p* = 0.004).

**Table 2.** Background characteristics for all participants and separately in men and women.

	Total (*n* = 454)	Women (*n* = 187)	Men (*n* = 267)	*p*-Value
Age (years), mean (SD)	62.5 (9.8)	62.9 (9.8)	62.1 (9.7)	0.395
Ethnicity, born in Sweden (%)	442 (97.4)	260 (97.4)	182 (97.3)	0.543
Proportion with BP <140/90 mmHg, *n* (%)	165 (36.3)	74 (39.6)	91 (34.1)	0.231
Office SBP (mmHg), mean (SD)	143.7 (16.2)	143.4 (16.7)	143.9 (15.9)	0.720
Office DBP (mmHg), mean (SD)	84.7 (8.9)	82.8 (8.4)	86.0 (9.1)	<0.001
Pulse, (beats/minute), mean (SD)	72.2 (12.1)	72.6 (11.6)	71.9 (12.5)	0.528
BMI (kg/m^2^), mean (SD, range)	28.8 (4.4)	28.8 (4.5)	28.9 (4.3)	0.825
HbA1c (mmol/mol), mean (SD)	38.8 (7.5)	38.3 (5.6)	39.1 (8.6)	0.313
eGFR (mL/min/1,73m^2^), mean (SD)	75.9 (13.7)	76.2 (14.2)	75.6 (13.4)	0.646
Total cholesterol (mmol/L), mean (SD)	5.0 (1.0)	5.3 (1.0)	4.8 (1.0)	<0.001
Hypertension duration (years), mean (SD)	9.6 (8.8)	10.3 (9.1)	9.1 (8.6)	0.178
Number of antihypertensive drugs, mean (SD)	1.5 (0.8)	1.4 (0.8)	1.6 (0.8)	0.126
Diuretics, *n* (%)	63 (13.9)	32 (17.1)	31 (11.6)	0.095
Beta-blockers, *n* (%)	108 (23.8)	50 (26.7)	58 (21.7)	0.217
Ca-antagonists, *n* (%)	160 (35.2)	47 (25.1)	113 (42.3)	<0.001
RAS blockers, *n* (%)	349 (76.9)	138 (73.8)	211 (79.0)	0.193
Other, *n* (%)	3 (0.7)	1 (0.5)	2 (0.7)	0.781
Diabetes, *n* (%)	57/453 (12.6)	20/186 (10.8)	37 (13.9)	0.327
Heart failure, *n* (%)	9/432 (2.1)	1/180 (0.6)	8/252 (3.2)	0.060
Current smoker, *n* (%)	23/447 (5.1)	8/184 (4.4)	15/263 (5.7)	0.523
Previous smoker, *n* (%)	115/447 (25.7)	46/184 (25.0)	69/263 (26.2)	0.769
Alcohol intake, standard drinks/week, *n* (%):				
<1	166/444 (37.4)	86/182 (47.3)	80/262 (30.5)	<0.001
1–9	251/444 (56.6)	92/182 (50.5)	159/262 (60.7)	0.034
>9	27/444 (6.1)	4/182 (2.2)	23/262 (8.9)	0.004
Marital status, *n* (%):				
Married or cohabiting	368/448 (82.1)	123/185 (66.5)	225/263 (85.6)	0.025
Unmarried or widowed	70/448 (15.6)	38/185 (20.5)	32/263 (12.2)	0.016
Other	10/448 (2.2)	4/185 (2.2)	6/263 (2.3)	0.933
Educational level, *n* (%):				
Less than high school	97/446 (21.7)	40/183 (21.9)	57/263 (21.7)	0.963
High school	203/446 (45.5)	67/183 (36.6)	136/263 (51.7)	0.002
University	146/446 (32.7)	76/183 (41.5)	70/263 (26.6)	<0.001
Occupation, *n* (%, several occupations possible):				
Working	236 (52.0)	82 (43.9)	154 (57.7)	0.004
Retired	222 (48.9)	105 (56.1)	117 (43.8)	0.010
Other (student or unemployed)	13 (2.9)	9 (4.8)	4 (1.5)	0.037
Physical activity, minutes/week (*n*, %):				
<30	223/442 (50.5)	88/182 (48.4)	135/260 (51.9)	0.460
30–89	119/442 (26.9)	47/182 (25.8)	72/260 (27.7)	0.663
≥90	100/442 (22.6)	47/182 (25.8)	53/260 (20.4)	0.179

*p*-Value for differences between men and women, calculated with student’s *t*-test for continuous data and pearson’s chi^2^-test for categorical data.

The results from the linear mixed effect models for SBP and DBP with variables as fixed effects and participants as random effects are presented in Table 3. Wellbeing, medication intake, headache, restlessness, physical activity, and self-reported stress were significantly associated with changes in both SBP and DBP. Separate linear mixed effect models for SBP and DBP were used for men and women and the results are presented in the online Supplementary material.

**Table 3.** Linear mixed-effect model for association between SBP and DBP and self-reported variables.

	Systolic BP				Diastolic BP			
Variables	Estimate	Standard error (SE)	*p-*Value	95% CI	Estimate	Standard error (SE)	*p*-Value	95% CI
Intercept	143.78	1.28	<0.001	141.26 − 146.29	81.84	0.84	<0.001	80.20 to 83.49
Wellbeing	−1.04	0.15	<0.001	−1.33 to −0.76	−0.41	0.10	<0.001	−0.60 to −0.22
Medication intake	−5.19	0.76	<0.001	−6.67 to −3.71	−2.63	0.49	<0.001	−3.60 to −1.66
Tiredness	−0.12	0.11	0.276	−0.35 to 0.10	−0.11	0.07	0.154	−0.25 to 0.04
Dizziness	−0.21	0.19	0.264	−0.57 to 0.16	−0.16	0.12	0.189	−0.40 to 0.08
Headache	0.38	0.15	0.010	0.09 to 0.68	0.39	0.10	<0.001	0.19 to 0.58
Palpitations	0.15	0.21	0.475	−0.26 to 0.56	0.16	0.14	0.255	−0.11 to 0.43
Restlessness	0.84	0.18	<0.001	0.49 to 1.19	0.42	0.12	<0.001	0.19 to0.65
Sleep	−0.02	0.10	0.884	−0.22 to 0.19	0.02	0.07	0.717	−0.11 to 0.16
Physical activity	−0.63	0.082	<0.001	−0.79 to −0.47	−0.13	0.05	0.012	−0.24 to −0.03
Stress	1.06	0.12	<0.001	0.83 to 1.30	0.59	0.08	<0.001	0.43 to 0.75

Self-reported stress and wellbeing affected the BP to a similar extent, with an increase of 1.1 mmHg for SBP and 0.6 mmHg for DBP for each level of more stress reported (*p* < 0.001), and a decrease in SBP of 1.0 mmHg and DBP of 0.4 mmHg for each level of higher wellbeing (*p* < 0.001). This accounts for an increase in SBP of 4.2 mmHg when stress is reported as ‘very much’ compared to ‘not at all’, and a decrease in SBP of 4.2 mmHg when wellbeing was reported ‘very bad’ compared to ‘very good’. The effect of stress on BP was similar for men and women, but the effect of wellbeing was lower for women for SBP and nonsignificant for DBP.

The greatest impact on BP in total, and for men and women separately, was seen in relation to medication adherence, with a decrease of 5.2 mmHg for SBP and 2.6 mmHg for DBP associated with taking the medication regularly (perfect adherence) compared to not taking the medication at all (non-adherence).

The associations were weaker but statistically significant for restlessness, headache and BP levels. Restlessness affected the BP levels more for women than for men. Headache was not significantly associated with SBP for women. Dizziness was only significantly associated with DBP for men.

Tiredness was significantly associated with BP levels for women (SBP *p* = 0.04, DBP *p* = 0.03), but not for the total study population. The effect on BP levels was lower than for other variables with significant associations.

Physical activity was associated with SBP reduction among men only, with a decrease of 0.89 mmHg for every self-reported step (*p* < 0.001).

The maximum possible number of observations per variable was 25,878 (454 participants × 57 days; the first day was numbered 0). In total, the number of observations per variable ranged from 22,239 to 22 569. On average, 13% of the observations were missing. The missing values were clustered to a few participants and increased slightly with time. The median number of daily self-reports (of at least one variable) was 53. The participants reported high adherence to medication treatment; 97.1% of the observations for medication intake were positive. In total, 346 participants (76.2%) consistently reported taking their medication throughout the intervention period.

## Discussion

### Principal findings

This observational study showed that self-reported higher wellbeing, lower restlessness and less stress, and higher medication adherence, were all associated with lower same-day BP levels. Physical activity was significantly associated with BP levels only for men, not for women. For the symptoms, headache was significantly associated with BP levels, but the association was weaker and nonsignificant for SBP for women.

### Strengths and weaknesses

This study is unique as it explores associations between daily home-monitored BP and same-day reports of quality of life, adherence to medication, lifestyle, and symptoms in a large cohort of patients with treated hypertension over a long period of time. This provides a rich dataset for analysis. The reporting rate was high; only 13% of the observations were missing. Since the study was conducted in primary health care, where most patients with hypertension in Sweden are treated, the results are highly relevant for hypertension management in clinical practice. The self-report system and the included variables are relevant and useful to patients with hypertension as it was developed and designed in collaboration with patients and professionals [13, 14].

As in all lifestyle intervention trials, there is a risk of recruitment bias. The patients who agreed to participate in the PERHIT trial could already be more motivated to make lifestyle changes and be interested in their own health than those who did not take part. The participants in our study reported very high adherence to medication, which is likely an effect of participating in a clinical trial and receiving daily reminders to self-report symptoms and measure BP. This weakness is, for obvious reasons, difficult to adjust for in a study such as this. Since the self-reports were made daily, the risk of recall bias is considered low. However, there is still a risk of participants forgetting if they took their medication in the morning, which might affect the reports on adherence to medication.

Another weakness of the study is that the participants were homogenous in terms of ethnicity, as 95% of the participants were born in Sweden. It is possible that the results would be different in a different ethnical population, and it would be of interest to test the self-management system in other ethnic groups.

### Findings in relation to other studies

In the material from the pilot study analysed and presented by Taft et al. [15], the results were similar to ours, with significant associations between lower BP and medication intake, better wellbeing, less stress, and more physical activity. The association between headache, restlessness and BP levels was not seen in the previous paper. The number of patients in the present study is almost 10 times higher and significant associations could therefore be seen where the absolute effect was smaller. As in the study by Taft et al. the highest impact on BP was, not surprisingly, seen in relation to medication adherence in this study. Our results confirm the conclusion drawn by Taft et al. that enabling persons with hypertension to gain first-hand knowledge of links between BP and their experienced quality of life, adherence, lifestyle, and symptoms may be a way to help them adhere to treatment.

We have previously described the results on BP control using the web-based system [12]. After eight weeks there was a significant effect on BP control, but one year after the intervention, the difference between the intervention and the control group was no longer significant and we can therefore not know if the effects of this self-management support system are sustainable. As described by Hellgren et al. [20], there is room for improvement in hypertension management in primary care in Sweden. There are several other important risk factors to cardiovascular disease, and although hypertension is the focus of this intervention, it is important to keep in mind that the overall risk for the individual should be prioritized in clinical care. By increased insight and motivation to healthy lifestyle habits, more risk factors than hypertension could be addressed. The web-based system used in this study could potentially be adjusted to suit high overall risk for cardiovascular disease. In future research, it might be of interest to explore how to best integrate the intervention in the shared decision-making process.

#### Quality of life

Psychosocial stress is considered a risk factor for hypertension and cardiovascular disease [21, 22]. Acute stress induces an increase in BP, but chronic stress has also been shown to cause long-lasting effects on BP [23]. In our study, SBP was estimated to be 4.2 mmHg higher when stress was reported as being high rather than low, with similar result in men and women. In the instructions to the participants, it was not specified what kind of stress the patients were to report. Occupational stress has been reported as being associated with increased BP and the development of hypertension [24]. This association appears to be stronger for men than for women [25, 26]. In our material, 44% of the women and 58% of the men worked, and therefore, other psychological stressors may also be involved for the participants. It may be reasonable to believe that stress and restlessness are experienced simultaneously, and that restlessness is like stress associated with BP levels, but to a somewhat lower extent. A high report of restlessness was associated with an increase in SBP of 3.4 mmHg compared to a low report of restlessness in our study.

Wellbeing was inversely related to BP levels, with the same effect on SBP as stress. A high rating of wellbeing was associated with an estimated decrease in SBP of 4.2 mmHg, compared to a low rating of wellbeing. There are some previous studies on how wellbeing is associated with BP. Low wellbeing was associated with higher BP levels in patients with hypertension and coronary artery disease in a study by Gong et al. [27]. In an American study, spiritual wellbeing was associated with lower BP levels in healthy religious persons [28]. On the contrary, low BP was associated with poor wellbeing in middle-aged men in a Swedish study by Rosengren et al. [29]. Wellbeing refers to the ‘evaluative judgements about selected aspects or the entirety of a life situation or life path’ [30], and was evaluated by the question ‘How do you feel today?’ in our study. Thus, our results support the hypothesis that stress and wellbeing influence BP levels and that the promotion of wellbeing and stress reduction may positively affect hypertension management. Future research may focus on how this knowledge may benefit patients with hypertension in clinical practice.

#### Symptoms

Hypertension is usually considered a symptomless condition, but there are studies showing that this is not how many patients experience the condition themselves [31, 32]. In a systematic review, Marshall et al. described that many patients believed that hypertension produced symptoms and based their decisions about taking or not taking medication on their experience of such symptoms [4]. In a recent systemic review, Horne et al. described a multitude of symptoms experienced by persons with hypertension [31]. Cantillon et al. reported that patients’ perceptions of blood pressure changes were not associated with BP levels [33]. In our study, headache was significantly associated with BP levels, but with a somewhat lower level of significance for SBP, and non-significant for women. Headache is commonly associated with hypertension by patients [31] but it is not concluded that mild to moderate hypertension causes headache [34]. However, various disorders with abrupt and severe increases in BP are associated with headache [31]. In hypertension guidelines, headache is mainly mentioned in the context of diagnoses causing secondary hypertension, or as a side effect of antihypertensive treatment [35]. Our results indicate that there may exist an association, between slightly increased BP levels and more headache. For the other symptoms reported in the study, dizziness was only significantly associated with DBP for men. Palpitations were neither associated with SBP nor DBP. Accordingly, patients’ beliefs about symptoms and BP levels are important to acknowledge and discuss during consultations for hypertension.

#### Physical activity

It is well known that physical activity has a positive impact on BP. Patients with hypertension can reduce their BP through regular physical activity [36]. Our results showed a significant association between lower BP levels and higher levels of physical activity, but only in men. The reported amount of time spent on physical activity did not differ significantly between men and women at baseline. As reported, some other baseline characteristics did, however, differ between sexes, but it is unlikely that this is the explanation for the different results. Some studies have shown that regular physical activity can reduce the risk of hypertension in women [37]. More research is needed on physical activity as a treatment for diagnosed hypertension in women.

## Conclusion

Daily life experiences and behaviour can be associated with BP levels in patients with hypertension. In clinical practice, it may be important to identify patients with high-stress levels and low wellbeing. The association between medication intake and BP was noticeable, stressing the importance of adherence to medication for patients with hypertension. Using a hypertension management system seems to be a promising tool for communication between the patient and physician, but we do not know if the effect on BP control is sustainable in the long run.

## Supplementary Material

Supplemental Material
