# Supplementary material for: Associations between daily home blood pressure measurements and self-reports of lifestyle and symptoms in primary care: the PERHIT study
Source: Scand J Prim Health Care. 2024 Mar 26;42(3):415–23. doi: 10.1080/02813432.2024.2332745 (PMC11332292; doi:10.1080/02813432.2024.2332745)
Supplement: Supplemental Material [file IPRI_A_2332745_SM8065.docx]

# BP and associations to self-reported variable for women and men

## Women

**Table 1.** Linear mixed-effect model for association between SBP, DBP and self-reported variables for women.

|  | **Systolic BP** | | | | **Diastolic BP** | | | |
| --- | --- | --- | --- | --- | --- | --- | --- | --- |
| **Variables** | **Estimate** | **Standard Error (SE)** | **P-value** | **95% CI** | **Estimate** | **Standard Error (SE)** | **P-value** | **95% CI** |
| *Intercept* | 137.35 | 1.86 | <0.001 | 133.67 - 141.01 | 77.77 | 1.24 | <0.001 | 75.33 - 80.21 |
| Wellbeing | -0.95 | 0.20 | <0.001 | -1.35 - -0.55 | -0.23 | 0.14 | 0.094 | -0.50 - 0.04 |
| Medication intake | -3.16 | 1.13 | 0.005 | -5.37 - -0.95 | -1.71 | 0.76 | 0.025 | -3.19 - -0.22 |
| Tiredness | -0.34 | 0.16 | 0.038 | -0.65 - -0.02 | -0.23 | 0.11 | 0.030 | -0.45 - -0.02 |
| Dizziness | 0.001 | 0.27 | 0.997 | -0.52 - 0.53 | 0.06 | 0.18 | 0.723 | -0.28 - 0.41 |
| Headache | 0.28 | 0.21 | 0.173 | -0.12 - 0.69 | 0.43 | 0.14 | 0.002 | 0.15 - 0.70 |
| Palpitations | 0.41 | 0.29 | 0.161 | -0.16 - 0.98 | -0.02 | 0.20 | 0.899 | -0.41 - 0.36 |
| Restlessness | 1.03 | 0.28 | <0.001 | 0.49 - 1.57 | 0.50 | 0.19 | 0.007 | 0.14 - 0.87 |
| Sleep | 0.003 | 0.14 | 0.982 | -0.28 - 0.28 | -0.03 | 0.10 | 0.786 | -0.22 - 0.17 |
| Physical activity | -0.24 | 0.12 | 0.057 | -0.48 - 0.01 | 0.08 | 0.08 | 0.345 | -0.09 - 0.24 |
| Stress | 1.11 | 0.17 | <0.001 | 0.78 - 1.45 | 0.65 | 0.12 | <0.001 | 0.43 - 0.88 |

## Men

**Table 2.** Linear mixed-effect model for association between SBP, DBP and self-reported variables for men.

|  | **Systolic BP** | | | | **Diastolic BP** | | | |
| --- | --- | --- | --- | --- | --- | --- | --- | --- |
| **Variables** | **Estimate** | **Standard Error (SE)** | **P-value** | **95% CI** | **Estimate** | **Standard Error (SE)** | **P-value** | **95% CI** |
| *Intercept* | 148.50 | 1.75 | <0.001 | 145.07 - 151.92 | 84.97 | 1.13 | <0.001 | 82.75 - 87.19 |
| Wellbeing | -1.14 | 0.21 | <0.001 | -1.55 - -0.73 | -0.60 | 0.13 | <0.001 | -0.86 - -0.34 |
| Medication intake | -6.72 | 1.01 | <0.001 | -8.70 - -4.74 | -3.35 | 0.65 | <0.001 | -4.62 - -2.07 |
| Tiredness | 0.09 | 0.16 | 0.580 | -0.22 - 0.39 | 0.02 | 0.10 | 0.812 | -0.17 - 0.22 |
| Dizziness | -0.38 | 0.26 | 0.138 | -0.89 - 0.12 | -0.37 | 0.17 | 0.025 | -0.70 - -0.05 |
| Headache | 0.48 | 0.21 | 0.024 | 0.06 - 0.89 | 0.35 | 0.14 | 0.010 | 0.09 - 0.62 |
| Palpitations | -0.03 | 0.30 | 0.917 | -0.61 - 0.55 | 0.35 | 0.19 | 0.066 | -0.02 - 0.73 |
| Restlessness | 0.68 | 0.24 | 0.004 | 0.22 - 1.14 | 0.33 | 0.15 | 0.028 | 0.04 - 0.63 |
| Sleep | -0.06 | 0.15 | 0.690 | -0.34 - 0.23 | 0.06 | 0.09 | 0.491 | -0.12 - 0.25 |
| Physical activity | -0.89 | 0.11 | <0.001 | -1.10 - -0.68 | -0.28 | 0.07 | <0.001 | -0.41 - -0.14 |
| Stress | 1.02 | 0.17 | <0.001 | 0.69 - 1.35 | 0.54 | 0.11 | <0.001 | 0.32 - 0.75 |
